# *N*-Benzoyl-*N*′-(1,10-phenanthrolin-5-yl)thio­urea dichloro­methane hemisolvate monohydrate

**DOI:** 10.1107/S160053681101734X

**Published:** 2011-05-14

**Authors:** Fatisha Liyana Mat Rashid, Lee Yook Heng, Jean-Claude Daran, Mohammad B. Kassim

**Affiliations:** a School of Chemical Sciences and Food Technology, Faculty of Science and Technology, Universiti Kebangsaan Malaysia, 43600 Bangi Selangor, Malaysia; b Laboratoire de Chimie de Coordination, UPR-CNRS 8241, 205 Route de Narbonne, F-31077 Toulouse CEDEX, France

## Abstract

The title compound, C_20_H_14_N_4_OS·0.5CH_2_Cl_2_·H_2_O, contains 1,10-phenanthroline and benzoyl fragments that adopt *cisoid* and *transoid* conformations respectively, with respect to the S atom. In the crystal, mol­ecules are linked by inter­molecular O—H⋯O, O—H⋯N, N—H⋯O and C—H⋯O hydrogen bonds, forming chains along [011]. Weak C—H⋯π and slipped π–π stacking inter­actions [centroid–centroid distances = 3.715 (3), 3.684 (3) and 3.574 (2) Å] are also observed. In addition to an ordered water mol­ecule of solvation, there is a disordered dichloro­methane solvent mol­ecule which was difficult to model correctly. The contributions to the electron density for this mol­ecule was removed using the *SQUEEZE* procedure in *PLATON* [Spek (2009 ▶). *Acta Cryst*. D**65**, 148–155].

## Related literature

For related structures, see: Al-abbasi & Kassim (2011 ▶); Hassan *et al.* (2008 ▶); Yamin & Hassan (2004 ▶); Yamin & Yusof (2003 ▶); Yunus *et al.* (2008 ▶). For standard bond lengths, see: Allen *et al.* (1987 ▶).
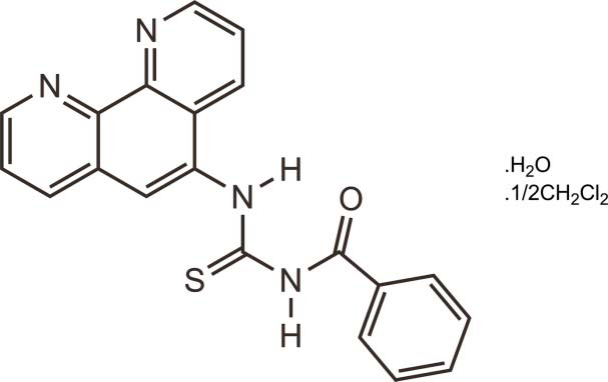


## Experimental

### 

#### Crystal data

C_20_H_14_N_4_OS·0.5CH_2_Cl_2_·H_2_O
*M* *_r_* = 418.89
Triclinic, 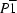

*a* = 9.385 (5) Å
*b* = 10.863 (5) Å
*c* = 10.927 (5) Å
α = 112.949 (5)°
β = 103.984 (5)°
γ = 96.641 (5)°
*V* = 967.6 (8) Å^3^
*Z* = 2
Mo *K*α radiation
μ = 0.33 mm^−1^
*T* = 298 K
0.47 × 0.19 × 0.14 mm

#### Data collection

Bruker SMART APEX CCD area-detector diffractometer
Absorption correction: multi-scan (*SADABS*; Bruker, 2000 ▶) *T*_min_ = 0.916, *T*_max_ = 0.974
4400 measured reflections
3254 independent reflections
2450 reflections with *I* > 2σ(*I*)
*R*_int_ = 0.014

#### Refinement

*R*[*F* ^2^ > 2σ(*F* ^2^)] = 0.049
*wR*(*F* ^2^) = 0.139
*S* = 1.08
3254 reflections
244 parameters
H-atom parameters constrained
Δρ_max_ = 0.28 e Å^−3^
Δρ_min_ = −0.19 e Å^−3^

### 

Data collection: *SMART* (Bruker, 2000 ▶); cell refinement: *SAINT* (Bruker, 2000 ▶); data reduction: *SAINT*; program(s) used to solve structure: *SHELXS97* (Sheldrick, 2008 ▶); program(s) used to refine structure: *SHELXL97* (Sheldrick, 2008 ▶); molecular graphics: *ORTEPIII* (Burnett & Johnson, 1996 ▶), *ORTEP-3 for Windows* (Farrugia, 1997 ▶) and *SHELXTL* (Sheldrick, 2008 ▶); software used to prepare material for publication: *SHELXTL*, *PARST* (Nardelli, 1995 ▶) and *PLATON* (Spek, 2009 ▶).

## Supplementary Material

Crystal structure: contains datablocks global, I. DOI: 10.1107/S160053681101734X/fj2403sup1.cif

Structure factors: contains datablocks I. DOI: 10.1107/S160053681101734X/fj2403Isup2.hkl

Additional supplementary materials:  crystallographic information; 3D view; checkCIF report

## Figures and Tables

**Table 1:** Hydrogen-bond geometry (Å, °) *Cg*2 and *Cg*3 are the centroids of the N4,C9–C12,C20 and C1–C6 rings, respectively.

*D*—H⋯*A*	*D*—H	H⋯*A*	*D*⋯*A*	*D*—H⋯*A*
N1—H1*A*⋯O1	0.86	1.96	2.636 (3)	135
N2—H2*A*⋯O2*W*	0.86	2.07	2.910 (3)	165
O2*W*—H1*W*⋯N3^i^	0.85	2.08	2.889 (3)	160
O2*W*—H2*W*⋯O1^ii^	0.85	2.46	3.187 (3)	144
C9—H9⋯O1^iii^	0.93	2.56	3.140 (4)	121
C2—H2⋯*Cg*2^iv^	0.93	2.99	3.806 (4)	147
C13—H13⋯*Cg*3^ii^	0.93	2.88	3.796 (3)	168

Symmetry codes: (i) 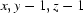; (ii) 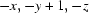; (iii) 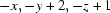; (iv) 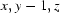.

**Fig. 1.**
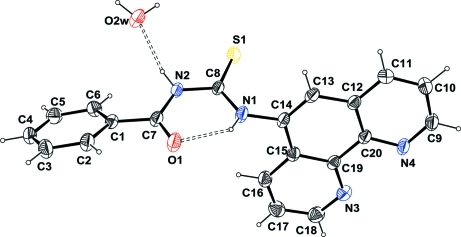
The molecular structure of (I) with the atom labeling scheme. Displacement ellipsoids are drawn at the 30% probability level. H atoms are represented as small spheres of arbitrary radii. Hydrogen bonds are shown as dashed lines.

**Fig. 2.**
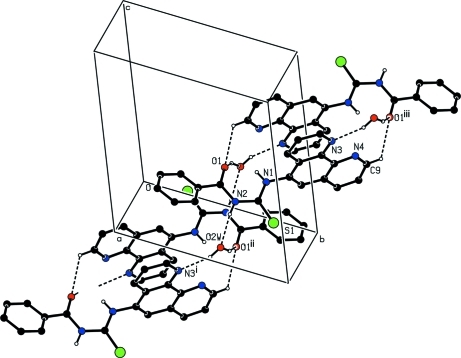
A partial packing view of (I) showing the O—H···O, O—H···N, N—H···O and C—H···O Hydrogen bonds whiwh are shown as dashed lines. H atoms attached to C atoms not involved in hydrogen bondings have been omitted for clarity.

## supplementary crystallographic information

### Comment

The title molecule maintains the *cis-trans* configuration with respect to the positions of the 1,10-phenanthrolin-5-amine and benzoyl groups, respectively, relative to the S atom across the C–N bonds (Fig 1). The bond lengths and angles in the molecules are in normal ranges (Allen *et al.*, 1987).

The phenyl and phenanthroline rings are twisted with relative to the central thiourea fragment dihedral angles of 29.46 (12)° and 74.06 (8)°, respectively. The phenyl and phenantroline rings are almost perpendicular to each other with dihedral angle of 83.15 (10)°.

An intramolecular N1—H1A···O1 (Fig. 1 and Table 2) stabilize the conformation and forms a pseudo six-membered ring (N1/H1A/O1/C7/N2/C8). The crystal packing is stabilized by fours intermolecular O2W—H2W···O1, O2W—H1W···N3, N2—H2A···O2W and C9—H9···O1 hydrogen bonds,(Table 1), which link the molecules into an infinite one-dimensional chain along [0 1 1] direction (Fig 2). There also exist weak C—H···π and slippest π-π stacking interactions which result in a three dimensionnal network.

### Experimental

The reaction scheme involved a reaction of benzoyl chloride (8.6 mmol) with ammonium thiocyanate (8.6 mmol) in acetone. The product, benzoyl isothiocyanate (7.7 mmol) was reacted with 1,10-phenanthrolin-5-amine (7.7 mmol) to give the title compound with a 70% yield. A slow evaporation dichloromethane solution of the product gave a colourless crystals suitable for X-ray diffraction.

### Refinement

All H atoms attached to C and N atoms were fixed geometrically and treated as riding with C—H = 0.93 Å and N—H = 0.86 Å with *U*_iso_(H) = 1.2*U*_eq_(C or N). H atoms of water molecule were located in difference Fourier maps and included in the subsequent refinement using restraints (O—H= 0.85 (1)Å and H···H= 1.39 (2) Å) with *U*_iso_(H) = 1.5*U*_eq_(O). In the last cycles of refinement, they were treated as riding on their parent O atom.

Some residual electron density were difficult to model and therefore, the SQUEEZE function of *PLATON* (Spek, 2009) was used to eliminate the contribution of the electron density in the solvent region from the intensity data, and the solvent-free model was employed for the final refinement. There is one cavity of 122.9 Å^3^ per unit cell. *PLATON* estimated that the cavity contains 38.4 electrons which may correspond to a solvent molecule of dichloromethane as suggested by chemical analyses.

This dichloromethane solvent has been however included in the formula.

### Crystal data

**Table:**

C_20_H_14_N_4_OS·0.5CH_2_Cl_2_·H_2_O	*Z* = 2
*M**_r_* = 418.89	*F*(000) = 434
Triclinic, *P*1	*D*_x_ = 1.438 Mg m^−^^3^
Hall symbol: -P 1	Mo *K*α radiation, λ = 0.71073 Å
*a* = 9.385 (5) Å	Cell parameters from 4905 reflections
*b* = 10.863 (5) Å	θ = 2.1–28.4°
*c* = 10.927 (5) Å	µ = 0.33 mm^−^^1^
α = 112.949 (5)°	*T* = 298 K
β = 103.984 (5)°	Block, colourless
γ = 96.641 (5)°	0.47 × 0.19 × 0.14 mm
*V* = 967.6 (8) Å^3^	

### Data collection

**Table:**

Bruker SMART APEX CCD area-detector diffractometer	3254 independent reflections
Radiation source: fine-focus sealed tube	2450 reflections with *I* > 2σ(*I*)
graphite	*R*_int_ = 0.014
ω scans	θ_max_ = 25.0°, θ_min_ = 2.1°
Absorption correction: multi-scan (*SADABS*; Bruker, 2000)	*h* = −11→10
*T*_min_ = 0.916, *T*_max_ = 0.974	*k* = −7→12
4400 measured reflections	*l* = −12→12

### Refinement

**Table:**

Refinement on *F*^2^	Primary atom site location: structure-invariant direct methods
Least-squares matrix: full	Secondary atom site location: difference Fourier map
*R*[*F*^2^ > 2σ(*F*^2^)] = 0.049	Hydrogen site location: inferred from neighbouring sites
*wR*(*F*^2^) = 0.139	H-atom parameters constrained
*S* = 1.08	*w* = 1/[σ^2^(*F*_o_^2^) + (0.0792*P*)^2^ + 0.0787*P*] where *P* = (*F*_o_^2^ + 2*F*_c_^2^)/3
3254 reflections	(Δ/σ)_max_ < 0.001
244 parameters	Δρ_max_ = 0.28 e Å^−^^3^
0 restraints	Δρ_min_ = −0.19 e Å^−^^3^

### Special details

**Table:**

Geometry. All e.s.d.'s (except the e.s.d. in the dihedral angle between two l.s. planes) are estimated using the full covariance matrix. The cell e.s.d.'s are taken into account individually in the estimation of e.s.d.'s in distances, angles and torsion angles; correlations between e.s.d.'s in cell parameters are only used when they are defined by crystal symmetry. An approximate (isotropic) treatment of cell e.s.d.'s is used for estimating e.s.d.'s involving l.s. planes.
Refinement. Refinement of *F*^2^ against ALL reflections. The weighted *R*-factor *wR* and goodness of fit *S* are based on *F*^2^, conventional *R*-factors *R* are based on *F*, with *F* set to zero for negative *F*^2^. The threshold expression of *F*^2^ > σ(*F*^2^) is used only for calculating *R*-factors(gt) *etc*. and is not relevant to the choice of reflections for refinement. *R*-factors based on *F*^2^ are statistically about twice as large as those based on *F*, and *R*- factors based on ALL data will be even larger.

### Fractional atomic coordinates and isotropic or equivalent isotropic displacement parameters (Å^2^)

**Table:**

	*x*	*y*	*z*	*U*_iso_*/*U*_eq_	
C8	0.1675 (2)	0.7038 (2)	0.1383 (2)	0.0355 (5)	
C7	0.1658 (3)	0.4881 (2)	0.1675 (2)	0.0392 (6)	
C14	0.0884 (3)	0.8891 (2)	0.3010 (2)	0.0376 (5)	
C13	−0.0219 (3)	0.9315 (2)	0.2377 (2)	0.0419 (6)	
H13	−0.0864	0.8715	0.1488	0.050*	
C12	−0.0432 (3)	1.0675 (2)	0.3038 (2)	0.0384 (5)	
C20	0.0499 (3)	1.1569 (2)	0.4405 (2)	0.0359 (5)	
C19	0.1718 (3)	1.1125 (2)	0.5081 (2)	0.0366 (5)	
C15	0.1916 (3)	0.9784 (2)	0.4377 (2)	0.0386 (5)	
C1	0.2387 (3)	0.3698 (2)	0.1377 (2)	0.0374 (5)	
C6	0.3666 (3)	0.3676 (3)	0.0951 (3)	0.0444 (6)	
H6	0.4046	0.4380	0.0751	0.053*	
C5	0.4371 (3)	0.2607 (3)	0.0825 (3)	0.0528 (7)	
H5	0.5231	0.2593	0.0544	0.063*	
C4	0.3802 (3)	0.1559 (3)	0.1116 (3)	0.0521 (7)	
H4	0.4284	0.0843	0.1034	0.063*	
C3	0.2526 (3)	0.1569 (3)	0.1525 (3)	0.0556 (7)	
H3	0.2136	0.0853	0.1704	0.067*	
C2	0.1833 (3)	0.2638 (2)	0.1669 (3)	0.0472 (6)	
H2	0.0983	0.2653	0.1965	0.057*	
C11	−0.1558 (3)	1.1162 (3)	0.2395 (3)	0.0490 (6)	
H11	−0.2178	1.0612	0.1482	0.059*	
C10	−0.1746 (3)	1.2442 (3)	0.3108 (3)	0.0533 (7)	
H10	−0.2479	1.2787	0.2689	0.064*	
C9	−0.0820 (3)	1.3217 (3)	0.4472 (3)	0.0506 (7)	
H9	−0.0988	1.4074	0.4964	0.061*	
C16	0.3117 (3)	0.9399 (3)	0.5063 (3)	0.0470 (6)	
H16	0.3276	0.8523	0.4638	0.056*	
C17	0.4052 (3)	1.0305 (3)	0.6353 (3)	0.0562 (7)	
H17	0.4865	1.0067	0.6814	0.067*	
C18	0.3767 (3)	1.1599 (3)	0.6968 (3)	0.0550 (7)	
H18	0.4411	1.2211	0.7852	0.066*	
N1	0.1038 (2)	0.75069 (19)	0.2384 (2)	0.0462 (5)	
H1A	0.0695	0.6940	0.2674	0.055*	
N2	0.1897 (2)	0.57097 (18)	0.1030 (2)	0.0384 (5)	
H2A	0.2221	0.5371	0.0325	0.046*	
N3	0.2644 (2)	1.2012 (2)	0.6378 (2)	0.0475 (5)	
N4	0.0287 (2)	1.2829 (2)	0.5128 (2)	0.0462 (5)	
O1	0.0911 (2)	0.51100 (18)	0.2479 (2)	0.0574 (5)	
S1	0.22322 (8)	0.79160 (7)	0.05756 (7)	0.0509 (2)	
O2W	0.2509 (2)	0.46575 (19)	−0.1633 (2)	0.0658 (6)	
H1W	0.2519	0.3976	−0.2352	0.099*	
H2W	0.1823	0.5024	−0.1909	0.099*	

### Atomic displacement parameters (Å^2^)

**Table:**

	*U*^11^	*U*^22^	*U*^33^	*U*^12^	*U*^13^	*U*^23^
C8	0.0397 (12)	0.0285 (11)	0.0331 (12)	0.0102 (9)	0.0112 (10)	0.0075 (9)
C7	0.0467 (14)	0.0288 (12)	0.0386 (13)	0.0088 (10)	0.0173 (11)	0.0089 (10)
C14	0.0492 (14)	0.0276 (11)	0.0418 (13)	0.0106 (10)	0.0272 (11)	0.0128 (10)
C13	0.0523 (15)	0.0341 (12)	0.0350 (13)	0.0085 (11)	0.0179 (11)	0.0087 (10)
C12	0.0489 (14)	0.0342 (12)	0.0376 (13)	0.0121 (10)	0.0224 (11)	0.0151 (11)
C20	0.0448 (13)	0.0286 (11)	0.0373 (13)	0.0088 (10)	0.0204 (10)	0.0129 (10)
C19	0.0430 (13)	0.0314 (11)	0.0378 (13)	0.0075 (10)	0.0205 (10)	0.0131 (10)
C15	0.0480 (14)	0.0357 (12)	0.0430 (13)	0.0132 (10)	0.0272 (11)	0.0195 (11)
C1	0.0448 (13)	0.0295 (11)	0.0347 (12)	0.0089 (10)	0.0132 (10)	0.0101 (10)
C6	0.0443 (14)	0.0456 (14)	0.0506 (15)	0.0134 (11)	0.0194 (12)	0.0247 (12)
C5	0.0499 (15)	0.0639 (17)	0.0500 (15)	0.0254 (13)	0.0205 (12)	0.0239 (14)
C4	0.0623 (17)	0.0432 (14)	0.0516 (16)	0.0266 (12)	0.0155 (13)	0.0187 (12)
C3	0.0677 (18)	0.0408 (14)	0.0679 (18)	0.0191 (13)	0.0272 (15)	0.0279 (14)
C2	0.0545 (15)	0.0352 (13)	0.0558 (16)	0.0126 (11)	0.0265 (13)	0.0176 (12)
C11	0.0552 (16)	0.0505 (15)	0.0403 (14)	0.0159 (12)	0.0124 (12)	0.0194 (12)
C10	0.0619 (17)	0.0511 (16)	0.0582 (17)	0.0276 (13)	0.0226 (14)	0.0289 (14)
C9	0.0629 (17)	0.0386 (13)	0.0566 (17)	0.0225 (12)	0.0263 (14)	0.0197 (13)
C16	0.0527 (15)	0.0452 (14)	0.0556 (16)	0.0205 (12)	0.0286 (13)	0.0250 (13)
C17	0.0478 (15)	0.0695 (19)	0.0607 (18)	0.0216 (14)	0.0183 (13)	0.0345 (16)
C18	0.0511 (16)	0.0588 (17)	0.0458 (15)	0.0110 (13)	0.0117 (12)	0.0160 (13)
N1	0.0699 (14)	0.0278 (10)	0.0503 (12)	0.0157 (9)	0.0354 (11)	0.0155 (9)
N2	0.0488 (12)	0.0315 (10)	0.0372 (11)	0.0155 (8)	0.0204 (9)	0.0112 (9)
N3	0.0485 (12)	0.0442 (12)	0.0432 (12)	0.0098 (10)	0.0151 (10)	0.0122 (10)
N4	0.0589 (13)	0.0325 (10)	0.0487 (12)	0.0184 (9)	0.0243 (10)	0.0125 (9)
O1	0.0855 (13)	0.0415 (10)	0.0681 (13)	0.0283 (9)	0.0510 (11)	0.0274 (9)
S1	0.0700 (5)	0.0451 (4)	0.0598 (4)	0.0279 (3)	0.0388 (4)	0.0303 (3)
O2W	0.0810 (14)	0.0525 (11)	0.0523 (11)	0.0243 (10)	0.0246 (10)	0.0067 (9)

### Geometric parameters (Å, °)

**Table:**

C8—N1	1.329 (3)	C5—H5	0.9300
C8—N2	1.395 (3)	C4—C3	1.375 (4)
C8—S1	1.651 (2)	C4—H4	0.9300
C7—O1	1.222 (3)	C3—C2	1.371 (4)
C7—N2	1.372 (3)	C3—H3	0.9300
C7—C1	1.486 (3)	C2—H2	0.9300
C14—C13	1.331 (3)	C11—C10	1.360 (4)
C14—N1	1.431 (3)	C11—H11	0.9300
C14—C15	1.437 (3)	C10—C9	1.383 (4)
C13—C12	1.432 (3)	C10—H10	0.9300
C13—H13	0.9300	C9—N4	1.322 (3)
C12—C11	1.397 (4)	C9—H9	0.9300
C12—C20	1.407 (3)	C16—C17	1.358 (4)
C20—N4	1.354 (3)	C16—H16	0.9300
C20—C19	1.444 (3)	C17—C18	1.391 (4)
C19—N3	1.355 (3)	C17—H17	0.9300
C19—C15	1.416 (3)	C18—N3	1.317 (3)
C15—C16	1.398 (3)	C18—H18	0.9300
C1—C6	1.388 (3)	N1—H1A	0.8600
C1—C2	1.388 (3)	N2—H2A	0.8600
C6—C5	1.379 (4)	O2W—H1W	0.8472
C6—H6	0.9300	O2W—H2W	0.8513
C5—C4	1.381 (4)		
			
N1—C8—N2	116.0 (2)	C5—C4—H4	119.8
N1—C8—S1	124.83 (17)	C2—C3—C4	119.8 (3)
N2—C8—S1	119.18 (17)	C2—C3—H3	120.1
O1—C7—N2	122.2 (2)	C4—C3—H3	120.1
O1—C7—C1	121.1 (2)	C3—C2—C1	120.7 (2)
N2—C7—C1	116.7 (2)	C3—C2—H2	119.7
C13—C14—N1	121.2 (2)	C1—C2—H2	119.7
C13—C14—C15	121.4 (2)	C10—C11—C12	119.8 (2)
N1—C14—C15	117.4 (2)	C10—C11—H11	120.1
C14—C13—C12	121.3 (2)	C12—C11—H11	120.1
C14—C13—H13	119.3	C11—C10—C9	118.3 (2)
C12—C13—H13	119.3	C11—C10—H10	120.8
C11—C12—C20	117.5 (2)	C9—C10—H10	120.8
C11—C12—C13	122.8 (2)	N4—C9—C10	124.8 (2)
C20—C12—C13	119.7 (2)	N4—C9—H9	117.6
N4—C20—C12	122.7 (2)	C10—C9—H9	117.6
N4—C20—C19	118.2 (2)	C17—C16—C15	119.9 (2)
C12—C20—C19	119.1 (2)	C17—C16—H16	120.0
N3—C19—C15	121.9 (2)	C15—C16—H16	120.0
N3—C19—C20	118.7 (2)	C16—C17—C18	118.5 (3)
C15—C19—C20	119.4 (2)	C16—C17—H17	120.7
C16—C15—C19	117.6 (2)	C18—C17—H17	120.7
C16—C15—C14	123.4 (2)	N3—C18—C17	124.2 (3)
C19—C15—C14	119.0 (2)	N3—C18—H18	117.9
C6—C1—C2	119.3 (2)	C17—C18—H18	117.9
C6—C1—C7	122.8 (2)	C8—N1—C14	124.39 (19)
C2—C1—C7	117.6 (2)	C8—N1—H1A	117.8
C5—C6—C1	119.9 (2)	C14—N1—H1A	117.8
C5—C6—H6	120.1	C7—N2—C8	127.66 (19)
C1—C6—H6	120.1	C7—N2—H2A	116.2
C6—C5—C4	120.0 (2)	C8—N2—H2A	116.2
C6—C5—H5	120.0	C18—N3—C19	117.9 (2)
C4—C5—H5	120.0	C9—N4—C20	116.8 (2)
C3—C4—C5	120.4 (2)	H1W—O2W—H2W	106.6
C3—C4—H4	119.8		

### Hydrogen-bond geometry (Å, °)

**Table:**

Cg2 and Cg3 are the centroids of the N4,C9–C12,C20 and C1–C6 rings, respectively.

**Table:**

*D*—H···*A*	*D*—H	H···*A*	*D*···*A*	*D*—H···*A*
N1—H1A···O1	0.86	1.96	2.636 (3)	135
N2—H2A···O2W	0.86	2.07	2.910 (3)	165
O2W—H1W···N3^i^	0.85	2.08	2.889 (3)	160
O2W—H2W···O1^ii^	0.85	2.46	3.187 (3)	144
C9—H9···O1^iii^	0.93	2.56	3.140 (4)	121
C2—H2···Cg2^iv^	0.93	2.99	3.806 (4)	147
C13—H13···Cg3^ii^	0.93	2.88	3.796 (3)	168

Symmetry codes: (i) *x*, *y*−1, *z*−1; (ii) −*x*, −*y*+1, −*z*; (iii) −*x*, −*y*+2, −*z*+1; (iv) *x*, *y*−1, *z*.

### Table 2 Table 2 π-π stacking interactions (Å,°)

Cg1 is the centroid of the C12—C20 ring.Cg2 is the centroid of the N4—C20 ringCg4 is the centroid of the N3—C19 ring

**Table:**

CgI	CgJ	CgI···CgJ^a^	α	CgI···P(J)^b^	CgJ···P(I)^c^	Slippage
Cg1	Cg2^v^	3.715 (3)	4.14	3.438	3.387	1.47 (mean value)
Cg1	Cg4^v^	3.684 (3)	1.64	3.395	3.352	1.48 (mean value)
Cg4	Cg4^v^	3.574 (2)	0.02	3.359	3.359	1.222

Symmetry codes: (v)-x,2-y,1-zNotes:a : Distance between centroidsb : Perpendicular distance of CgI on ring plan Jc : Perpendicular distance of CgJ on ring plan Iα    = Dihedral Angle between the ring planes Slippage = vertical displacement between ring centroids.
